# Gene Correction Recovers Phagocytosis in Retinal Pigment Epithelium Derived from Retinitis Pigmentosa-Human-Induced Pluripotent Stem Cells

**DOI:** 10.3390/ijms22042092

**Published:** 2021-02-20

**Authors:** Ana Artero-Castro, Kathleen Long, Andrew Bassett, Almudena Ávila-Fernandez, Marta Cortón, Antonio Vidal-Puig, Pavla Jendelova, Francisco Javier Rodriguez-Jimenez, Eleonora Clemente, Carmen Ayuso, Slaven Erceg

**Affiliations:** 1 Stem Cells Therapies in Neurodegenerative Diseases Lab, Centro de Investigación Principe Felipe (CIPF), 46012 Valencia, Spain; aartero@cipf.es (A.A.-C.); frodriguez@cipf.es (F.J.R.-J.); eclemente@cipf.es (E.C.); 2 Wellcome Sanger Institute, Wellcome Genome Campus, Hinxton, Cambridge CB10 1SA, UK; kl16@sanger.ac.uk (K.L.); ab42@sanger.ac.uk (A.B.); 3 Department of Genetics and Genomics, IIS-Fundación Jiménez Díaz, (IIS-FJD, UAM), 28040 Madrid, Spain; AAvila@quironsalud.es (A.Á.-F.); mcorton@quironsalud.es (M.C.); CAyuso@fjd.es (C.A.); 4 Center for Biomedical Network Research on Rare Diseases (CIBERER), ISCIII, 28040 Madrid, Spain; 5 Metabolic Research Laboratories, Wellcome Trust MRC Institute of Metabolic Science, University of Cambridge, Addenbrooke’s Hospital, Cambridge CB2 0QQ, UK; ajv22@medschl.cam.ac.uk; 6 Institute of Experimental Medicine, Department of Neuroregeneration, Czech Academy of Science, 14220 Prague, Czech Republic; pavla.jendelova@iem.cas.cz; 7 National Stem Cell Bank-Valencia Node, Proteomics, Genotyping and Cell Line Platform, PRB3, ISCIII, Research Centre Principe Felipe, c/ Eduardo Primo Yúfera 3, 46012 Valencia, Spain

**Keywords:** induced pluripotent stem cells, Retinitis Pigmentosa, RPE, gene correction

## Abstract

Hereditary retinal dystrophies (HRD) represent a significant cause of blindness, affecting mostly retinal pigment epithelium (RPE) and photoreceptors (PRs), and currently suffer from a lack of effective treatments. Highly specialized RPE and PR cells interact mutually in the functional retina, therefore primary HRD affecting one cell type leading to a secondary HRD in the other cells. Phagocytosis is one of the primary functions of the RPE and studies have discovered that mutations in the phagocytosis-associated gene Mer tyrosine kinase receptor (*MERTK*) lead to primary RPE dystrophy. Treatment strategies for this rare disease include the replacement of diseased RPE with healthy autologous RPE to prevent PR degeneration. The generation and directed differentiation of patient-derived human-induced pluripotent stem cells (hiPSCs) may provide a means to generate autologous therapeutically-relevant adult cells, including RPE and PR. However, the continued presence of the *MERTK* gene mutation in patient-derived hiPSCs represents a significant drawback. Recently, we reported the generation of a hiPSC model of MERTK-associated Retinitis Pigmentosa (RP) that recapitulates disease phenotype and the subsequent creation of gene-corrected RP-hiPSCs using Clustered Regularly Interspaced Short Palindromic Repeats (CRISPR)/Cas9. In this study, we differentiated gene-corrected RP-hiPSCs into RPE and found that these cells had recovered both wild-type MERTK protein expression and the lost phagocytosis of fluorescently-labeled photoreceptor outer segments observed in uncorrected RP-hiPSC-RPE. These findings provide proof-of-principle for the utility of gene-corrected hiPSCs as an unlimited cell source for personalized cell therapy of rare vision disorders.

## 1. Introduction

Retinitis pigmentosa (RP), as a heterogeneous group of diseases, represents a common type of hereditary retinal dystrophy (HRD), causing blindness with a prevalence of ~1 in 2500 births and affecting more than one million people worldwide [1]. The causative mutations in HRD mainly affect highly specialized cells whose interactions are fundamental to retinal function: retinal pigment epithelium (RPE) and photoreceptors (PR). RPE is composed of a monolayer of pigmented cells that lies close to the photoreceptor outer segment (POS) and plays a crucial role in retinal homeostasis. Monogenic primary RPE dystrophies include mutations in genes, such as *lecithin-retinol acetyltransferase* (*LRAT*) [2], *RPE65* [3], *BESTROPHIN* (*BEST1*) [4], and *Mer tyrosine kinase receptor* (*MERTK*) [5]. Importantly, studies have established that if a primary HRD affects RPE cells, a secondary dystrophy usually arises in the other cells, such as PR [5,6].

The reprogramming of human somatic cells into induced pluripotent stem cells (human-induced pluripotent stem cells (hiPSCs)) has enormous potential in the field of disease modeling and regenerative medicine [7]. RPE-related HRDs represent promising candidates for modeling and cell therapy using hiPSCs, thanks to the development of high-yield RPE differentiation protocols (recently reviewed in Reference [8]).

Of note, autologous cell transplantation for RP bears the disadvantage of carrying the same genetic predisposition; therefore, causative mutations must be corrected in the case of hereditary dystrophies. The CRISPR (Clustered Regularly Interspaced Short Palindromic Repeats)/Cas9 system has already proven a rapid, simple, and effective method for gene-editing [9] and has been applied in hiPSCs generated from RP patients, including for the correction of the point mutation that causes X-linked RP [10].

Cases of RP caused by mutations in the *Mertk* gene, initially described in the Royal College of Surgeons (RCS) rat [11], result in an autosomal recessive form of blindness characterized by impaired phagocytosis of POS by RPE. *MERTK* gene mutations lead to the expression of a truncated protein that unable to phagocyte POS by RPE [5,12]. The subsequent accumulation of POS debris represents a possible mechanism of retinal degeneration and RP in humans [13].

In a previous study, we modeled human early-onset RP caused by a novel mutation in the human *MERTK* gene (homozygous frameshift mutation c.992_993delCA(p.Ser331Cysfs*5)) using hiPSC technology [12]. This in vitro model faithfully recapitulated the disease phenotype described in animals and patients, including the abolition of POS phagocytosis. We recently applied CRISPR/Cas9 editing to the previously generated RP-hiPSC line (RP1-FiPS4F1) and created and characterized two gene-corrected RP-hiPSC lines, RP1-FiPS4F1-GC1 and RP1-FiPS4F1-GC2, with one or both mutant alleles corrected, respectively [14].

In this study, we report the differentiation of gene-corrected RP-hiPSC into RPE that expresses full-length MERTK protein and displays wild-type-like levels of phagocytosis in vitro when both alleles are corrected. Overall, we believe that gene-correction of patient-derived hiPSCs and subsequent generation into functional RPE represents a platform for the development of cell therapies for MERTK-related HRD.

## 2. Results

### 2.1. Correction of RP-associated MERTK Gene Mutation

Previously generated the RP1-FiPS4F1 hiPSC line with *MERTK* gene mutation (RP-hiPSC) [12] and two resultant genetically corrected cell lines using CRISPR/Cas9 editing (gene-corrected RP-hiPSCs) with one or both mutant alleles corrected (RP1-FiPS4F1-GC1 and RP1-FiPS4F1-GC2, respectively) [14] are expanded as individual clones and subjected direct genomic sequencing to confirm genotype (Figure S1).

### 2.2. Differentiation of hiPSCs into RPE

**Figure 1:**
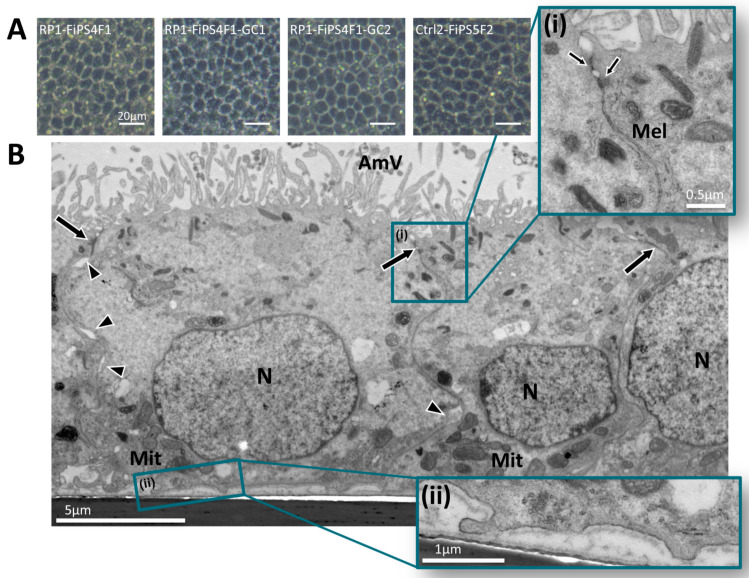
Differentiation of human-induced pluripotent stem cells (hiPSCs) into retinal pigment epithelium (RPE). (**A**) Representative brightfield micrograph of RPE derived from the four hiPSC lines employed in this study. All hiPSC-RPE show typical RPE polygonal morphology and pigmentation. (**B**) Representative electron micrograph of cultured RPE from the corrected hiPSC line (RP1-FiPS4F1-GC2). RPE form a monolayer of cuboid cells highly polarized with abundant apical microvilli (AmV) and melanosomes (Mel). The intercellular junctional complexes include apical tight junctions and adherens junctions (arrows), as well as membrane interdigitations (arrowheads). The nuclei and (N) and mitochondria (Mit) are located on the basal side of the cells. (**i**) High magnification shows melanin granules (Mel) and cell-cell junctions (arrows) (**ii**) High magnification of the basement membrane of the cells tightly bound to the transwells support film and small basal infoldings. Scale bars: A 20 µm; B 5 µm, (**i**) 0.5 µm, (**ii**) 1 µm.

We induced the differentiation of hiPSC lines into RPE using modified protocol of Brandl et al. [15] (see methods) (Figure S2). There were no differences in differentiation efficiency between the hiPSC lines. After approximately 1 month, pigmented cell clusters started to spontaneously appear in confluent hiPSC culture (Figure S2A–B). Subsequent to the first passage to enrich RPE cells, the hiPSC-derived RPE exhibited the polygonal, cobblestone-like morphology characteristic of mature RPE after three to five weeks in culture (Figure S2C). At this stage, we lifted cells and reseeded them onto permeable culture inserts to yield a uniform and polarized cellular monolayer. To reach full functional maturity, we cultured cells for at least two months until cultures displayed polygonal morphology and pigmentation (Figure 1A). Ultrastructural studies using transmission electron microscopy (TEM) confirmed that cell monolayers of mature RPE derived from four hiPSC lines displayed the correct apical location of microvilli, cilia, and cell-cell junctions (tight and adherents junctions) (Figure 1B, representative image of RPE from RP1-FiPS4F1-GC2). We observed cell nuclei located on the basal side of the hiPSC-RPE monolayer and ellipsoidal mitochondria below the nuclei at the basal-lateral part of the cells (Figure 1B). Intercellular junctional complexes, including tight junctions, adherens junctions (Figure 1B, arrows), and membrane interdigitations (Figure 1B, arrowheads), are visible in appropriately aligned sections of all three types of hiPSC-RPE revealing the integrity and function of the RPE monolayer. Other cellular structures, such as melanosomes, black round and oval shapes in detail in Figure 1(Bi), displayed an apical distribution. Furthermore, the basement membrane of the cells is visible with small basal infoldings (Figure 1(Bii)). These observations demonstrate that hiPSC-RPE grown in culture inserts form well-organized monolayers with the correct localization and polarization of key RPE ultrastructures.

**Figure 2:**
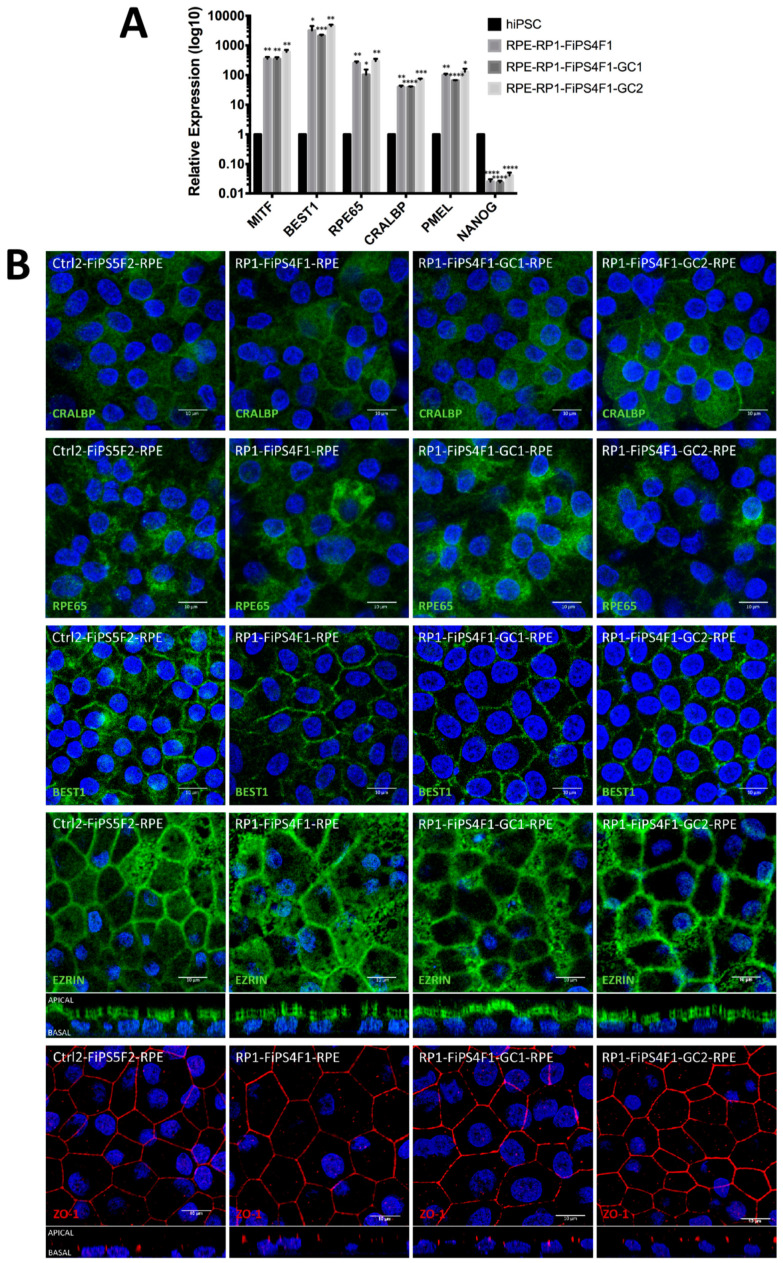
RPE-associated gene and protein expression in hiPSC-RPE. (**A**) qRT-PCR analysis of mRNA expression for RPE markers (*MITF*, *BESTROPHIN* (*BEST1)*, *RPE65*, *CRALBP*, and *PMEL*) and pluripotency marker *NANOG*. Data represent fold-change in mRNA expression of three hiPSC-RPE lines relative to their hiPSC. Each bar represents the average ±SEM of at least three independent biological replicates. The differences in mRNA expression between the hiPSC-RPE and their originating hiPSC were tested for significance (* *p*  ≤  0.05, ** *p*  ≤  0.01, *** *p*  ≤  0.001, **** *p*  ≤  0.0001). (**B**) Immunocytochemistry analysis of protein expression for RPE markers (CRALBP, RPE65, BEST1, EZRIN, and Zonula Occludens protein-1 (ZO-1)) in hiPSC-RPE. Representative confocal z-stacks micrographs of polarized RPE for apical expression of EZRIN and apico-lateral expression of ZO-1. Images were taken with Leica confocal microscope TCS SP8 (Wetzlar, Germany) using HCX PL APO lambda blue 63X/ 1.4 oil objective. Scale bar 10 µm.

To validate the identity of RP-hiPSC-RPE and gene-corrected RP-hiPSC-RPE, we carried out gene expression analysis (qRT-PCR) for RPE-specific genes (Figure 2A). All RPE demonstrated significantly higher expression for all RPE specific markers: *MICROPHTHALMIA-ASSOCIATED TRANSCRIPTION FACTOR (MITF)*, *BESTROPHIN 1* (*BEST1*), *RPE65*, *CELLULAR RETINALDEHYDE-BINDING PROTEIN (CRALBP)* and *PMEL*, compared to their parental hiPSCs, while expression of pluripotency marker *NANOG* exhibited a reduction in expression in RPE, as expected. Overall, we establish that RP-hiPSC-RPE and gene-corrected RP-hiPSC-RPE expressed genes typical of mature RPE.

Protein expression analysis performed by immunocytochemical staining revealed the robust expression of the CRALBP and RPE65 as visual cycle markers, BEST1, the microvilli marker EZRIN, and the tight junction marker Zonula Occludens protein-1 (ZO-1) in RP-hiPSC-RPE and gene-corrected RP-hiPSC-RPE, similar to RPE derived from control hiPSCs (Figure 2B). The apico-lateral and apical localization of ZO-1 and EZRIN, respectively, are both hallmarks of RPE and we discovered appropriate ZO-1 and EZRIN localization in RP-hiPSC-RPE and gene-corrected RP-hiPSCs-RPE.

### 2.3. MERTK Expression in hiPSC-RPE

**Figure 3:**
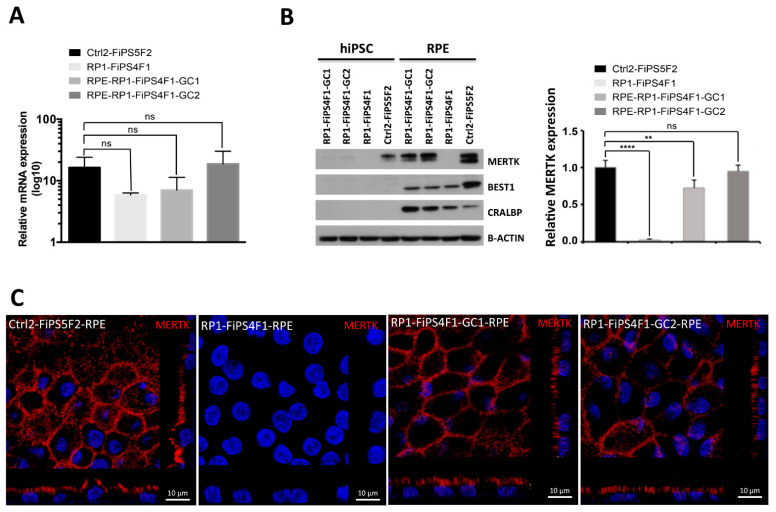
Recovery of Mer tyrosine kinase receptor (MERTK) expression in vitro. (**A**) qRT-PCR analysis of mRNA expression for *MERTK.* Data represent fold-change in mRNA expression of four hiPSC-RPE lines relative to their hiPSC. Each bar represents the average ±SEM of at least three independent biological replicates. The differences in mRNA expression between the control hiPSC-RPE with the uncorrected and corrected RP-hiPSC-RPE were tested for significance. (**B**) Western blot analysis of RPE-specific marker protein expression in hiPSC-RPE. The expression of CRALBP and BEST1 is detected in hiPSC-RPE, but not in the respective parental hiPSCs. MERTK expression is detected in corrected and control hiPSC-RPE but not in uncorrected RP-hiPSC-RPE. β-ACTIN used as loading control. The graph shows the relative quantification of MERTK expression representative of three different experiments of western blot. The differences in protein expression between the control hiPSC-RPE with the uncorrected and corrected RP-hiPSC-RPE were tested for significance (** *p*  ≤  0.01, **** *p*  ≤  0.0001) (**C**) MERTK expression in hiPSC-RPE. Confocal z-stacks micrographs show apical MERTK distribution in gene-corrected RP-hiPSC-RPE (RP1-FiPS4F1-GC1 and RP1-FiPS4F1-GC2) and healthy control hiPSC-RPE (Ctrl2-FiPS5F2). We failed to detect MERTK expression in uncorrected RP-hiPSC-RPE (RP1-FiPS4F1).

We perform comparative expression analysis of *MERTK* at mRNA level by qRT-PCR in RPE, normalized to their respective parental hiPSCs (Figure 3A). All RPE cells lines express *MERTK* with no statistically significant differences, but this expression seems to be lower in uncorrected and one allele corrected RP-hiPSC-RPE, possibly because eukaryotes possess a nonsense-mediated mRNA decay pathway, which degrades mRNAs containing nonsense mutations before they are translated into nonfunctional polypeptides [16].

To analyze the *MERTK* expression at protein level, we used an antibody that detects the N-terminal fragment of the MERTK implying that any truncated protein retaining the N-terminal domain is absent in RP-hiPSC-RPE^12^. However, full MERTK protein in corrected and control will be detected by this antibody. We compared MERTK and mature RPE specific markers by Western blot in RPE to their respective parental hiPSCs (Figure 3B). We observed similar expression levels of the RPE-specific markers BEST1 and CRALBP in all RPE monolayer samples and a lack of expression in undifferentiated hiPSCs, further confirming RPE phenotype. MERTK is detected as a double band that migrates with molecular weights of 180 and 130 kDa in both gene-corrected RP-hiPSC-RPE and control hiPSC-RPE while being absent in RP-hiPSC-RPE harboring the MERTK mutation, as noted in our previous study [12]. We detected weak MERTK expression in all undifferentiated corrected RP-hiPSCs, as well as control hiPSCs, upon higher exposure, but not in RP-hiPSC (Supplementary Figure S3), a phenomenon already described and discussed in our previous study [12] and elsewhere [17]. The relative quantification of MERTK protein expression reveals differences when compared uncorrected and one allele corrected RP-hiPSC-RPE with control hiPSC-RPE, but there are no differences between two alleles corrected and control hiPSC-RPE (Figure 3B). Additional immunocytological staining for MERTK in RPE monolayers confirmed the expression of full MERTK protein, as well as correct apical localization in gene-corrected and control hiPSC-RPE, while it was absent in RP-hiPSC-RPE (Figure 3C).

### 2.4. Recovery of Phagocytosis in RPE Derived from Gene-Corrected RP-hiPSCs

**Figure 4:**
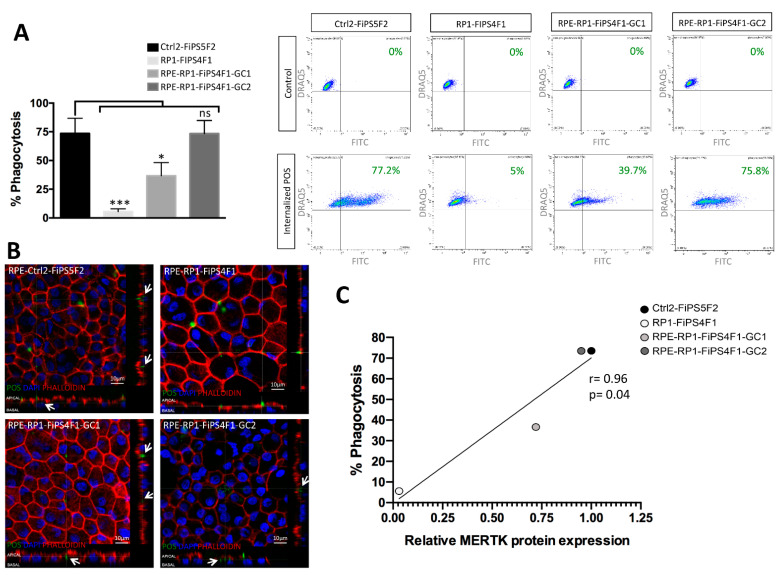
Recovery of photoreceptor outer segments (POS) phagocytosis shown by in vitro phagocytosis assay of POS in hiPSC-RPE. (**A**) Quantification of phagocytosis assay by flow cytometry. The graph represents the percentage of phagocyte cells of four hiPSC-RPE lines relative to their control (cells without incubation with POS-FITC). Each bar represents the average ±SEM of at least three independent biological replicates. The differences in % of phagocytes between the hiPSC-RPE were tested for significance (* *p* ≤ 0.05, *** *p* ≤ 0.001). The plots shown the results of one experiment. Cells with internalized POS-FITC (phagocytes) are placed in the upper right quadrant (DRAQ5 and FITC positive cells). (**B**) Confocal images of phagocytosis assay show basolateral sections across the hiPSC-RPE together with vertical section simulation. The control hiPSC-RPE and corrected RP-hiPSC-RPE internalize FITC labeled POS (green) (arrows), while the uncorrected RP-hiPSC-RPE do not. F-actin is stained by phalloidin (red) to visualize cell boundary. Images were taken with Leica confocal microscope TCS SP8 using HCX PL APO lambda blue 63X/ 1.4 OIL objective, scale bar 10 μm. (**C**) The Pearson correlation analysis was calculated to evaluate the link between relative MERTK protein expression and the percentage of RPE cells that phagocyte, and there is a significant and strong positive linear correlation between both parameters (r = 0.96; *p* = 0.04).

The process of POS phagocytosis can be stimulated in cultured RPE via incubation with isolated bovine-derived POS [12,18]. We employed this system (using fluorescently labeled bovine POS) and monitored ingestion by RPE via confocal imaging. Encouragingly, gene-corrected RP-hiPSC-RPE regained their ability to phagocytose POS (Figure 4B); phalloidin-labeling depicts abundant F-actin in the microvilli, and we detected POS in the microvilli region and inside RPE derived from gene-corrected and control hiPSCs (Figure 4B). Figure 4B depicts vertical sectioning demonstrating the ingested POS within phagosomes of RPE (arrows). As expected, we failed to observe the internalization of POS by RP-hiPSC-RPE, which showed only a few POS in the area of microvilli. We used flow cytometry to quantify the phagocytosis assay, and we obtained similar percentage of phagocytes in RPE derived from two allele gene-corrected and control hiPSCs (73.4 and 73.5%, respectively), which was double that observed in RPE with one allele gene-corrected (36.7%) (Figure 4A). Interestingly, we observed that there is a direct correlation between MERTK protein expression and percentage of the RPE cells undergoing phagocytosis of POS. We performed correlative analysis of these 2 parameters and found that there is a significant (*p* = 0.04) and strong positive linear correlation (r = 0.96) between MERTK protein expression and percentage of RPE cells that phagocyte POS (Figure 4C).

## 3. Discussion

In this study, we demonstrate the successful differentiation of gene-corrected RP-hiPSC derived from a patient carrying the mutation *p*.Ser331Cysfs*5 that displayed complete deficiency in MERTK function. At the same time, we demonstrated the reestablishment of the expression of full-length MERTK protein, as well as the reversion of lost phagocyte function of RP-hiPSC-RPE in vitro, which represents the first example of its kind in this field. We used the genetically corrected patient-derived hiPSC in which one or both alleles were corrected, as well as patient’s RP-hiPSC and control healthy hiPSC, which were all differentiated toward RPE cells. All generated RPE cells have many of the features of primary RPE cells; they grow as epithelial monolayer with a cobblestone morphology, with apical pigmented melanosomes, basal nuclei and express proteins involved in the process of retinol cycling. All corrected RPE cells, as well as control RPE and the RP-RPE, express the *MERTK* mRNA as evidenced by qRT-PCR, but this expression seems to be lower in uncorrected and one allele corrected RP-hiPSC-RPE. This difference in expression was more pronounced at the protein level with total absence of detectable MERTK protein in the patient’s RPE. Gene correction of one allele or both alleles was able to restore expression of MERTK that was not present in the uncorrected patient-derived cells. However, we observed a significant difference in MERTK expression between RPE derived from one or two alleles corrected. The restoration of protein expression correlates into a functional rescue. Partial restoration of MERTK level in RPE derived from one allele corrected hiPSC is in direct correlation to partial phagocytosis capacity. One explanation for this is that the partially restored protein expression is not able to trigger downstream molecules involved in phagocytosis resulting in slower phagocytic rate. Since MERTK receptor density is critical another explanation could be that an altered balance of MERTK expression dramatically affects the dynamics of RPE cells to phagocyte POS [19]. It is debatable whether the gene correction in only one allele and improvement of approx. 40% of phagocyte cells would be enough in vivo to slow the progression of the disease in future cell therapy. Only fully established MERTK expression leads to fully functional restoration similar to control cells, suggesting that only fully recovered protein may facilitate a functional improvement in vivo. RPE-related dystrophies are at the forefront of cell-based therapy development due to their simple connectivity, spacious subretinal space, and the ability to monitor injected cells by Optical coherence tomography (OCT). RPE transplants have proven successful, human embryonic stem cells (hESC) [20] or hiPSC derived RPE [21] in age-related macular degeneration patients, resulting in different outcomes. The generation of patient-specific stem cell-based tissue-engineered RPE may provide a unique platform for autologous cell replacement therapy and disease model development.

CRISPR/Cas9-mediated gene-editing has successfully corrected mutations associated with various ophthalmic pathologies [22], opening the possibility to create healthy patient-specific cells for transplantation. MERTK mutations account for approximately 1% of all RP cases and have been reported in several families with retinal dystrophy. The individuals that harbor mutations in this gene are affected with severe and progressive retinal disease, likely resulting from defective phagocytosis by RPE [13].

Recently, the results of a gene therapy trial involving the AAV2-mediated delivery of MERTK provided controversial outcomes [23]. Another potential therapeutic strategy is to target MERTK-related RP using small molecules; translational read-through inducing drugs (TRIDs) stimulate the bypass of the premature stop codon allowing continuation of translation, restoring a full-length MERTK protein [24], but this strategy can be used only in case of mutations creating stop codon. However, various drawbacks of this strategy highlight the requirement for new gene editing strategies to treat these genetic disorders. Although recent publication of Suzuki et al. [25] shows that in vivo correction of mutation causing RP is possible, the approach did not restore completely vision in animal model.

The generation of gene-corrected RPE from hiPSCs derived from patients with the *MERTK* mutation, as shown in this study, has several potential advantages over gene therapy, pharmacological therapy with TRIDS, or allogenic hiPSC-based RPE therapy. Our strategy permits the elimination of immunosuppressive therapy in the case of cell strategies that use healthy allogenic RPE, avoids the risk of insertional mutagenesis by therapeutic vectors, and maintains expression by endogenous control elements rather than constitutive promoters. Targeted genome manipulation at the endogenous genetic locus ensures the appropriate temporal and tissue-specific expression of the affected gene under the regulation of endogenous elements and is, thus, an attractive method for therapies or disease modeling.

## 4. Conclusions

Our findings confirm that normal cell phenotype can be re-established in patient-derived cells in vitro by genetic correction. Functional studies in vivo in animal models, such as RCS rats or larger animals, will confirm whether this strategy can completely restore the vision and be employed as a potential treatment for patients, taking into account that MERTK-related RP develops in early childhood when the therapeutic interventions are significantly reduced compared to other forms of RP.

## 5. Material and Methods

### 5.1. Maintenance of hiPSCs

Cell lines employed in this study included previously generated hiPSCs derived from patient-derived fibroblasts (RP1-FiPS4F1) [12] that were gene-corrected using the CRISPR/Cas9 system in which one allele or two alleles were corrected (RP1-FiPS4F1-GC1 and RP1-FiPS4F1-GC2, respectively) [14]. After single cell subcloning under feeder-free conditions, cell lines were fully characterized [14]. Cells were maintained on human embryonic stem cell (hESC)-qualified Matrigel (BD Biosciences, East Rutherford, NJ, USA) in mTeSR1 medium (StemCell Technologies, Vancouver, BC, Canada). Medium was changed daily and enzymatically passaging with dispase (1 mg/mL, StemCell Technologies) every 5–7 days at splitting ratios of 1:6 to 1:15. As a control, we used hiPSC derived from a healthy subject (Ctrl2-FiPS5F2) previously generated in our laboratory [26].

### 5.2. Mutation Sequencing

Genomic DNA from hiPSCs was isolated using the QIAamp DNA Blood mini kit (Qiagen, Hilden, Germany). Primers used for amplification and directed sequencing of *MERTK* upstream and downstream of c.992_993delCA were as follows: 5´CGAAGAGGTTCTAAGAGAGG3´ and 5´CCATTTTCATCAGTCGCCTC3´ (annealing temperature 55 ºC).

### 5.3. Differentiation toward RPE

To obtain RPE, hiPSC colonies were expanded to confluence in mTeSR1 maintenance medium (StemCell Technologies) for 7-10 days. At this point (day 0), differentiation was induced by switching hiPSC maintenance medium to RPE medium containing knockout DMEM, 20% knockout serum, Glutamax 2 mM (Invitrogen, Carlsbad, CA, USA), 0.1 mM non-essential amino acids, 0.23 mM β-mercaptoethanol (TermoFisher, MA, USA), 100 U/mL penicillin, 0,1 mg/mL streptomycin, and 10mM nicotinamide (Sigma-Aldrich, San Luis, Mi, USA) (Supplementary Figure S2). The medium was changed daily. Pigmented cell clusters appeared in culture within 3 to 4 weeks and enlarged progressively during the following 3 to 4 weeks (Supplementary Figure S2B). Pigmented patches were manually excised with a scalpel, enzymatically dissected into single cells by Tryp-LE select (Gibco), and replated on growth factor-reduced (GFR)-Matrigel (dilution 1:30)-coated cell culture plates in RPE medium (passage 1). Depending on cell confluency, 3 to 5 weeks later, monolayers of cells with RPE characteristic morphology (polygonal shape) appeared (Figure S2C). Medium was changed every other day, and passaging was performed every 3 to 4 weeks until passage five in which the cells reach senescence. For passages 2–5, the cells were detached with 1 mM EDTA in PBS (TermoFisher, Waltham, MA, USA) for 10 min followed by Tryp-LE select for 10 min and split at ratios from 1:6 to 1:10 on GFR- Matrigel-coated plates in RPE medium. For the analysis, we used cells with less than four passages.

For characterization and functional analysis, RPE was passed through a 40 μm strainer and plated at 200,000 cells/cm^2^ on GFR- Matrigel-coated transwell inserts (0.4 μm pore, Corning) to encourage polarized growth.

### 5.4. Phagocytosis Assays

Isolated bovine POS were obtained from InVision BioResources (WA, USA). For POS labeling, we used 2 mg/mL stock solution of FITC isomer 1 (F7250, Sigma) in 0.1 M sodium bicarbonate at pH 9, prepared, filter-sterilized, and stored in aliquots at −20 °C. For labeling with FITC, POS were suspended in a solution containing 10% sucrose, 20 mM sodium phosphate at pH 7.2, and 5 mM taurine and incubated with FITC for 1.5 h at room temperature, rotating in the dark. FITC-labeled POS were washed 3–5 times in labeling solution, resuspended in DMEM, counted, and stored at −80 °C until use in 2.5% sucrose DMEM to a concentration of 5 × 107 POS-FITC/mL.

To perform the phagocytosis assay, the hiPSC-RPE were cultured on transwell inserts for at least two months and incubated with POS-FITC for 3 h at 37 °C in 5% CO2 protected from light in RPE medium + 10% Fetal Bovine Serum (FBS), applying a concentration of 20 POS per cell. Then, the non-incorporated POS were removed by triple washing with PBS. After performing the phagocytosis assay, we use these cells for qualitative confocal imaging analysis or quantitative flow cytometry analysis.

To obtain the confocal images, after the non-incorporated POS were removed by triple washing, cells were fixed by 4% paraformaldehyde (PFA) for 15 min/RT, washed and permeabilized by 0.1% Triton-X. Phalloidin staining was performed for 30 min; the samples were then mounted with VectaShield mounting media (Vector Lab, Burlingame, CA, USA) with 4′,6- Diamidino-2-Phenylindole, Dihydrochloride (DAPI). The samples were visualized by Leica confocal microscope TCS SP8 using HCX PL APO lambda blue 63X/ 1.4 oli objective and analyzed by z-stacks to show the internalized OS with a green fluorescent marker (FITC).

To develop a quantitative phagocytosis assay, we used flow cytometry as reported previously [19] with minor modifications: cells were incubated with POS-FITC as described above and then, the non-incorporated POS were removed by triple washing with 1X Dulbecco’s PBS (with Calcium and Magnesium; Invitrogen). Untreated cells were used to obtain baseline fluorescence. To detect only the internalized POS, the cells were incubated with FITC-quenching solution (0.4% trypan blue in PBS) for 10 min at room temperature. After FITC-quenching, the cells were washed with PBS and treated with 1 mM EDTA in PBS for 10 min followed by Tryp-LE select for 10 min to detach cells and release bound POS. DMEM/F12 (Invitrogen) containing 2% FBS (Invitrogen) was added to neutralize the Tryp-LE and cells were transferred to flow cytometry tubes. Samples were diluted 1:1 with FACS staining buffer containing DRAQ5 (1:2500; Cell Signaling) and 2% FBS in PBS, and incubate 5 min at RT. Cells were labeled with DRAQ5 before analysis to distinguish cells from debris and POS. Propidium iodide (1ug/mL; Sigma) was added to evaluate cell viability, and the samples were analyzed immediately on CytoFLEX flow cytometer (Beckman Coulter, Brea, CA, USA). The live cells were gated based on DRAQ5 labeling, and 10,000 events were collected per sample. Data from at least three biological replicates were analyzed using CytExpert software (version 2.2.0.97; Beckman Coulter, Inc).

### 5.5. Transmission Electron Microscopy

After fixation in 4% paraformaldehyde, 2% glutaraldehyde in 0.1 M sodium phosphate buffer (pH 7,2–7,4) for 2 h, RPE cultured cells were washed with the same buffer, and then post-fixed in 1% OsO_4_ in phosphate buffer. Gradual dehydration were performed in ethanol series and the pieces were embedded in EPON 812. Leica DMR light microscope (Leica Microsystems) was used to analyze semi-thin sections which were stained with 1% toluidine blue in 3% sodium tetra-borate. Semi-thin and ultrathin sections were obtained in an ultramicrotome (Leica Ultracut R, Leica Microsystem.). After staining with lead citrate and uranyl acetate, JEM-1400 Plus electron microscope (JEOL GmbH, München, Germany) was used to examine the ultrathin sections.

### 5.6. RNA Extraction and Quantitative Real-Time PCR (qRT-PCR)

Cells were harvested and collected by centrifugation. Total RNA was isolated with the RNeasy Mini Kit (Qiagen, Hilden, Germany) following the manufacturer´s instructions. The samples were than treated with DNase1 to remove any genomic DNA contamination. cDNA synthesis from 1 μg of total RNA were performed using QuantiTect Reverse Transcription Kit (Qiagen) according to the manufacturer´s instructions. For quantitative real-time PCR (qRT-PCR), the relative quantification analysis was performed using the LightCycler 480 System (Roche, Basil, Switzerland). The PCR cycling program consisted of denaturing at 95 °C for 10 min followed by 40 cycles of 95 °C for 15 s and annealing/elongation at 60 °C for 1 min. The reactions were done in triplicate using TaqMan Gene Expression Master Mix. The list of TaqMan probes (Applied Biosystems, Foster City, CA, USA) is shown in Supplementary Table S1. To check the expression of *MERTK* we used qPCR probe that spans exon junction 18–19 corresponding to the tyrosine kinase functional domain. PCR was done in triplicate, and the expression of polymerase 2A (POL2A; Hs00172187_m1) was used as endogenous control to normalize the variations in cDNA quantities from different samples. The results were analyzed using LightCycler 480 software.

### 5.7. Immunocytochemistry

Cells were washed in PBS and fixed in 4% paraformaldehyde for 15 min. Fixed cells were washed twice in PBS and placed in blocking solution (3% normal goat serum and 0.5% Triton-X100 in PBS) for 1 h at room temperature. Cells were then incubated overnight at 4 °C with primary antibody. The following day, cells were washed three to five times in PBS and incubated with an appropriate secondary antibody (1:500, Invitrogen). After secondary antibody incubation, nuclei were stained with 4′,6- Diamidino-2-Phenylindole, Dihydrochloride (DAPI) (TermoFisher, MA, USA, #D1306), washed three times in PBS. Samples grown on transwell inserts were mounted using VectaShield Mounting Medium (Vector Lab, Burlingame, CA, USA) and imaged on Leica confocal microscope TCS SP8 using HCX PL APO lambda blue 63X/ 1.4 OIL objective and analyzed by z-stacks to show the polarized expression. The antibodies used are listed in Supplementary Table S2. To check the expression of *MERTK*, we used an antibody that detects the N-terminal fragment of the protein.

### 5.8. Western Blot Analysis

RPE or hiPSC were lysed in RIPA buffer (R0278 Sigma-Aldrich) containing a protease inhibitor mix (GE Healthcare), and total protein was quantified using a Bradford Reagent protein assay (B6916 Sigma-Aldrich). Protein lysates were denatured by 1X SDS Sample Buffer (70607 Novagen Sigma-Aldrich, San Luis, MI, USA). The resulting samples were incubated at 97 ºC for five minutes. Protein samples (30–50 µg) were then separated on TGX Stain-FreeTM Gels (Bio-Rad, Hercules, CA, USA), visualized by Molecular Image Gel DocTM XR (Bio-Rad) and electroblotted onto a PVDF membrane (Trans-Blot^®^ TurboTM Transfer Pack/ Bio-Rad). Membranes were incubated in blocking buffer (Prod#37515 TermoFisher, MA, USA) for 45 min to 1 h at room temperature, washed twice in TBS + 0.1% Tween for 5 min and incubated with primary antibody in blocking buffer overnight at 4 °C, except for β-ACTIN antibody (2 h at room temperature). Thereafter, blots were washed five times in TBS + 0.1% Tween and incubated with secondary antibody in blocking buffer for 45 min at room temperature. Blots were washed another five times, and protein bands were visualized using WesternBrightTM ECL (Advansta, San Jose, CA, USA). Mouse and rabbit secondary antibodies were obtained from Sigma and used at a concentration of 1:20,000. The primary antibodies used are listed in Supplementary Table S2. To check the expression of *MERTK*, we used an antibody that detects the N-terminal fragment of the protein. The relative MERTK protein quantification of the Western blot results of at least three independent experiments were determined by densitometry using Image J software. The values were normalized to β-ACTIN and then compared to the control group, which was normalized as 1.

### 5.9. Statistical Methods

Data are shown as mean ± SEM of at least three biological replicates and statistical analysis was calculated by unpaired t-test to determine differences in gene expression, one way ANOVA followed by Tukey’s multiple comparison test to analyze differences between control hiPSC-RPE versus corrected and uncorrected RP-hiPSC-RPE, as well as Pearson correlation coefficient to determine the correlation between MERTK protein expression and percentage of the RPE cells undergoing phagocytosis of POS, using GraphPad Prism 6 (GraphPad Software, San Diego, CA, USA). The differences were significant when *p* < 0.05 (* *p*  ≤  0.05, ** *p*  ≤  0.01, *** *p*  ≤  0.001, **** *p*  ≤  0.0001).

## Data Availability

Data sharing is not applicable to this article as no new data were created or analyzed in this study.

## Supplementary Materials

Supplementary materials can be found at https://www.mdpi.com/1422-0067/22/4/2092/s1.
