# Supplementary material for: Gene Correction Recovers Phagocytosis in Retinal Pigment Epithelium Derived from Retinitis Pigmentosa-Human-Induced Pluripotent Stem Cells
Source: Int J Mol Sci. 2021 Feb 20;22(4):2092. doi: 10.3390/ijms22042092 (PMC7923278; doi:10.3390/ijms22042092)
Supplement: Supplementary file 1 [file ijms-22-02092-s001.zip › ijms-1090241/1090241-SI.docx]

**Supplementary methods**

Mutation sequencing

Genomic DNA from hiPSCs was isolated using the QIAamp DNA Blood mini kit (Qiagen, Hilden, Germany). Primers used for amplification and directed sequencing of *MERTK* upstream and downstream of c.992_993delCA were as follows: 5´CGAAGAGGTTCTAAGAGAGG3´ and 5´CCATTTTCATCAGTCGCCTC3´ (annealing temperature 55 °C).

Differentiation toward RPE

To obtain RPE, hiPSC colonies were expanded to confluence in mTeSR1 maintenance medium (StemCell Technologies) for 7–10 days. At this point (day 0), differentiation was induced by switching hiPSC maintenance medium to RPE medium containing knockout DMEM, 20% knockout serum, Glutamax 2 mM (Invitrogen), 0.1 mM non-essential amino acids, 0.23 mM β-mercaptoethanol (Gibco), 100 U/ml penicillin, 0,1 mg/ml streptomycin, and 10mM nicotinamide (Sigma) (**Supplementary Fig. 2**). The medium was changed daily. Pigmented cell clusters appeared in culture within 3 to 4 weeks and enlarged progressively during the following 3 to 4 weeks (**Supplementary Fig. 2B**). Pigmented patches were manually excised with a scalpel, enzymatically dissected into single cells by Tryp-LE select (Gibco), and replated on growth factor-reduced (GFR-) Matrigel (dilution 1:30)-coated cell culture plates in RPE medium (passage 1). Depending on cell confluency, 3 to 5 weeks later, monolayers of cells with RPE characteristic morphology (polygonal shape) appeared (**Supplementary Fig. 2C**). Medium was changed every other day, and passaging was performed every 3 to 4 weeks until passage five in which the cells reach senescence. For passages 2-5, the cells were detached with 1 mM EDTA in PBS (Gibco) for 10 min followed by Tryp-LE select for 10 min and split at ratios from 1:6 to 1:10 on GFR- Matrigel-coated plates in RPE medium. For the analysis we used cells with less than four passages.


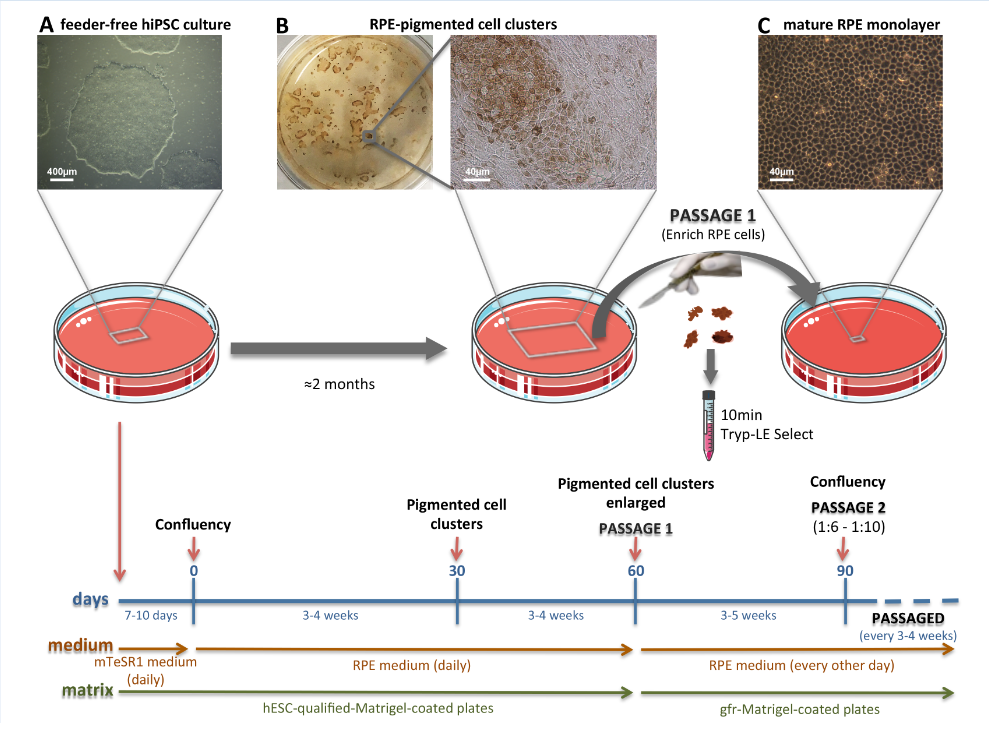


For characterization and functional analysis, RPE was passed through a 40 μm strainer and plated at 200,000 cells/cm^2^ on GFR- Matrigel-coated transwell inserts (0.4 μm pore, Corning) to encourage polarized growth.

Phagocytosis assays

Isolated bovine POS were obtained from InVision BioResources (WA, USA). For POS labeling we used 2 mg/ml stock solution of FITC isomer 1 (F7250, Sigma) in 0.1 M sodium bicarbonate at pH 9, prepared, filter-sterilized, and stored in aliquots at −20 °C. For labeling with FITC, POS were suspended in a solution containing 10% sucrose, 20 mM sodium phosphate at pH 7.2, and 5 mM taurine and incubated with FITC for 1.5 h at room temperature, rotating in the dark. FITC-labeled POS were washed 3-5 times in labeling solution, resuspended in DMEM, counted, and stored at −80 °C until use in 2.5% sucrose DMEM at a concentration of 5 × 10^7^ POS-FITC/ml.

hiPSC-RPE cultured on transwell inserts for at least two months were incubated with POS-FITC for 3 h at 37 °C in 5% CO_2_ protected from light in RPE medium containing 10% FBS, applying a concentration of 20 POS per cell. Then, the non-incorporated POS were removed by triple washing with PBS. Cells were fixed by 4% PFA for 15 min/RT, washed and permeabilized by 0.1% Triton-X. Phalloidin-TRITC staining (P1951, Sigma) was performed for 30 min; the samples were then mounted with VectaShield mounting media with DAPI. The samples were visualized by Leica confocal microscope TCS SP8 using HCX PL APO lambda blue 63X/ 1.4 OIL objective and analyzed by z-stacks to show the internalized POS with a green fluorescent marker (FITC).

To develop a quantitative phagocytosis assay we used flow cytometry as reported previously^1^ .Cells were incubated with POS-FITC as described above and then, the non-incorporated POS were removed by triple washing with 1X Dulbecco's PBS (with Calcium and Magnesium; Invitrogen). Untreated cells were used to obtain baseline fluorescence. To detect only the internalized POS, the cells were incubated with FITC-quenching solution (0.4% trypan blue in PBS) for 10 min at room temperature. After FITC-quenching, the cells were washed with PBS and treated with 1 mM EDTA in PBS for 10 min followed by Tryp-LE select for 10 min to detach cells and release bound POS. DMEM/F12 (Invitrogen) containing 2% FBS (Invitrogen) was added to neutralize the Tryp-LE and cells were transferred to flow cytometry tubes. Samples were diluted 1:1 with FACS staining buffer containing DRAQ5 (1:2500; Cell Signaling) and 2% FBS in PBS, and incubated 5min at RT. Cells were labeled with DRAQ5 before analysis to distinguish cells from debris and POS. Propidium iodide (1ug/ml; Sigma) was added to evaluate cell viability and the samples were analyzed immediately on CytoFLEX flow cytometer (Beckman Coulter). The live cells were gated based on DRAQ5 labeling, and 10000 events were collected per sample. Data from at least three biological replicates were analyzed using CytExpert software (version 2.2.0.97; Beckman Coulter, Inc).

Transmission electron microscopy

RPE cultured cells were fixed in 4% paraformaldehyde, 2% glutaraldehyde in 0.1 M sodium phosphate buffer (pH 7.2–7.4) for 2 h, washed with the same buffer, and then post-fixed in 1% OsO_4_ in PB. After gradual dehydration in ethanol series, the pieces were embedded in EPON 812. Semi-thin sections stained with 1% toluidine blue in 3% sodium tetra-borate were analyzed with a Leica DMR light microscope (Leica Microsystems). Semi-thin and ultrathin sections were obtained in an ultramicrotome (Leica Ultracut R, Leica Microsystem.). After staining with lead citrate and uranyl acetate, ultrathin sections were examined in a JEM-1400 Plus electron microscope (JEOL GmbH, München, Germany).

RNA extraction and quantitative real-time PCR (qRT-PCR)

Cells were collected by centrifugation, and total RNA was isolated with the RNeasy Mini Kit (Qiagen, Hilden, Germany) following the manufacturer´s instructions, and treated with DNase1 to remove any genomic DNA contamination. QuantiTect Reverse Transcription Kit (Qiagen) was used to carry out cDNA synthesis from 1 μg of total RNA according to the manufacturer´s instructions. For quantitative real-time PCR (qRT-PCR), the relative quantification analysis was performed using the LightCycler 480 System (Roche). The PCR cycling program consisted of denaturing at 95 °C for 10 minutes followed by 40 cycles of 95 °C for 15 seconds and annealing/elongation at 60 °C for 1 min. The reactions were done in triplicate using TaqMan Gene Expression Master Mix. The list of TaqMan probes (Applied Biosystems, Foster City, CA, USA) is shown in **Supplementary Table S1**. To check the expression of MERTK we used qPCR probe that spans exon junction 18–19 corresponding to the tyrosine kinase functional domain. PCR was done in triplicate, and the expression of polymerase 2A (POL2A; Hs00172187_m1) was used as endogenous control to normalize the variations in cDNA quantities from different samples. The results were analyzed using LightCycler 480 software and Microsoft Excel.

**Scheme 1.** TaqMan gene expression assay probes.

| **Gene** | **Assay ID** |
| --- | --- |
| RPE65 | Hs01071462_m1 |
| MITF | Hs01117294_m1 |
| BEST1 | Hs00188249_m1 |
| CRALBP | Hs00165632_m1 |
| MERTK | Hs01031973_m1 |
| PMEL | Hs00173854_m1 |
| NANOG | Hs02387400_g1 |
| POL2A | Hs00172187_m1 |

Immunocytochemistry

Cells were washed in PBS and fixed in 4% paraformaldehyde for 15 minutes. Fixed cells were washed twice in PBS and placed in blocking solution (3% normal goat serum and 0.5% Triton-X100 in PBS) for 1 h at room temperature. Cells were then incubated overnight at 4ºC with primary antibody. The following day, cells were washed three to five times in PBS and incubated with an appropriate secondary antibody (1:500, Invitrogen). After secondary antibody incubation, nuclei were stained with 4',6- Diamidino-2-Phenylindole, Dihydrochloride (DAPI) (Life Technologies, #D1306), washed three times in PBS. Samples grown on transwell inserts were mounted using VectaShield Mounting Medium (Vector Lab, Burlingame, CA, USA) and imaged on Leica confocal microscope TCS SP8 using HCX PL APO lambda blue 63X/ 1.4 OIL objective and analyzed by z-stacks to show the polarized expression. The antibodies used are listed in **Supplementary Table S2**. To check the expression of MERTK we used an antibody that detects the N-terminal fragment of the protein.

**Table2.** Antibodies used for western blot (WB) and immunocytochemistry (ICC).

| **Antibody** | **Origin** | **Reference** | **Dilution** | **Size (KDa)** |
| --- | --- | --- | --- | --- |
| MERTK | Rabbit | Abcam (ab52968) | WB 1:500  ICC 1:50 | 180-130 |
| BEST1 | Rabbit | Abcam (ab14928) | WB 1:500 | 68 |
|  | Mouse | Novus (NB300-164) | ICC 1:100 |  |
| CRALBP | Mouse | Abcam (ab15051) | WB 1:1000  ICC 1:100 | 36-40 |
| β-ACTIN-(Peroxidase) |  | Sigma (A3854) | WB 1:400.000 | 42 |
| RPE65 | Mouse | Millipore (MAB5428) | ICC 1:100 |  |
| EZRIN | Mouse | Sigma (E8897) | ICC 1:100 |  |
| ZO-1 | Rabbit | Invitrogen (617300) | ICC 1:50 |  |
| PHALLOIDIN-(TRITC) |  | Sigma (P1951) | ICC 50 mg/m |  |

Western blot analysis

RPE or hiPSC were lysed in RIPA buffer (R0278 Sigma) containing a protease inhibitor mix (GE Healthcare), and total protein was quantified using a Bradford Reagent protein assay (B6916 Sigma-Aldrich). Protein lysates were denatured by 1× SDS Sample Buffer (70607 Novagen). The resulting samples were incubated at 97 °C for five minutes. Protein samples (30–50 μg) were then separated on TGX Stain-FreeTM Gels (Bio-Rad), visualized by Molecular Image Gel DocTM XR (Bio-Rad) and electroblotted onto a PVDF membrane (Trans-Blot® TurboTM Transfer Pack/ Bio-Rad). Membranes were incubated in blocking buffer (Prod#37515 Thermo Scientific) for 45 min to 1 h at room temperature, washed twice in TBS + 0.1% Tween for 5 min and incubated with primary antibody in blocking buffer overnight at 4 °C, except for β-ACTIN antibody (2 h at room temperature). Thereafter, blots were washed five times in TBS + 0.1% Tween and incubated with secondary antibody in blocking buffer for 45 min at room temperature. Blots were washed another five times, and protein bands were visualized using WesternBrightTM ECL (Advansta). Mouse and rabbit secondary antibodies were obtained from Sigma and used at a concentration of 1:20,000. The primary antibodies used are listed in **Supplementary Table S2**. To check the expression of MERTK we used an antibody that detects the N-terminal fragment of the protein. The relative MERTK protein quantification of the Western blot results of at least three independent experiments were determined by densitometry using Image J software. The values were normalized to β-ACTIN and then compared to the control group, which was normalized as 1.

Statistical methods

Data are shown as mean±SEM of at least three biological replicates and statistical analysis was calculated by unpaired t-test to determine differences in gene expression, one way ANOVA followed by Tukey´s multiple comparison test to analyze differences between control hiPSC-RPE vs. corrected and uncorrected RP-hiPSC-RPE, and Pearson correlation coefficient to determine the correlation between MERTK protein expression and percentage of the RPE cells undergoing phagocytosis of POS, using GraphPad Prism 6 (GraphPad Software). The differences were significant when *p* < 0.05 (* *p*  ≤  0.05, ** *p*  ≤  0.01, *** *p*  ≤  0.001, **** *p*  ≤  0.0001).

Reference

1. Westenskow P.D.; Moreno, S.K., Krohne, T.U.; Kurihara,T.; Zhu, S.; Zhang, Z.-N.; Zhao, T.; Xu, Y.; Ding, S.; Friedlander, M. Using flow cytometry to compare the dynamics of photoreceptor outer segment phagocytosis in iPS-derived RPE cells. Investig. Ophthalmol. Vis. Sci*.* **2012**, *53*, 6282­6290.
