# Supplementary material for: Gene Correction Recovers Phagocytosis in Retinal Pigment Epithelium Derived from Retinitis Pigmentosa-Human-Induced Pluripotent Stem Cells
Source: Int J Mol Sci. 2021 Feb 20;22(4):2092. doi: 10.3390/ijms22042092 (PMC7923278; doi:10.3390/ijms22042092)
Supplement: Supplementary file 1 [file ijms-22-02092-s001.zip › ijms-1090241/Supplementary Table S1.docx]

**Supplementary Table S1.** TaqMan gene expression assay probes.

| **Gene** | **Assay ID** |
| --- | --- |
| RPE65 | Hs01071462_m1 |
| MITF | Hs01117294_m1 |
| BEST1 | Hs00188249_m1 |
| CRALBP | Hs00165632_m1 |
| MERTK | Hs01031973_m1 |
| PMEL | Hs00173854_m1 |
| NANOG | Hs02387400_g1 |
| POL2A | Hs00172187_m1 |
