# Supplementary material for: Gene Correction Recovers Phagocytosis in Retinal Pigment Epithelium Derived from Retinitis Pigmentosa-Human-Induced Pluripotent Stem Cells
Source: Int J Mol Sci. 2021 Feb 20;22(4):2092. doi: 10.3390/ijms22042092 (PMC7923278; doi:10.3390/ijms22042092)
Supplement: Supplementary file 1 [file ijms-22-02092-s001.zip › ijms-1090241/Supplementary Table S2.docx]

**Supplementary Table S2.** Antibodies used for western blot (WB) and immunocytochemistry (ICC).

| **Antibody** | **Origin** | **Reference** | **Dilution** | **Size (KDa)** |
| --- | --- | --- | --- | --- |
| MERTK | Rabbit | Abcam (ab52968) | WB 1:500  ICC 1:50 | 180-130 |
| BEST1 | Rabbit | Abcam (ab14928) | WB 1:500 | 68 |
|  | Mouse | Novus (NB300-164) | ICC 1:100 |  |
| CRALBP | Mouse | Abcam (ab15051) | WB 1:1000  ICC 1:100 | 36-40 |
| β-ACTIN-(Peroxidase) |  | Sigma (A3854) | WB 1:400.000 | 42 |
| RPE65 | Mouse | Millipore (MAB5428) | ICC 1:100 |  |
| EZRIN | Mouse | Sigma (E8897) | ICC 1:100 |  |
| ZO-1 | Rabbit | Invitrogen (617300) | ICC 1:50 |  |
| PHALLOIDIN-(TRITC) |  | Sigma (P1951) | ICC 50 mg/m |  |
