# Supplementary figures and images for: Gene Correction Recovers Phagocytosis in Retinal Pigment Epithelium Derived from Retinitis Pigmentosa-Human-Induced Pluripotent Stem Cells
Source: Int J Mol Sci. 2021 Feb 20;22(4):2092. doi: 10.3390/ijms22042092 (PMC7923278; doi:10.3390/ijms22042092)

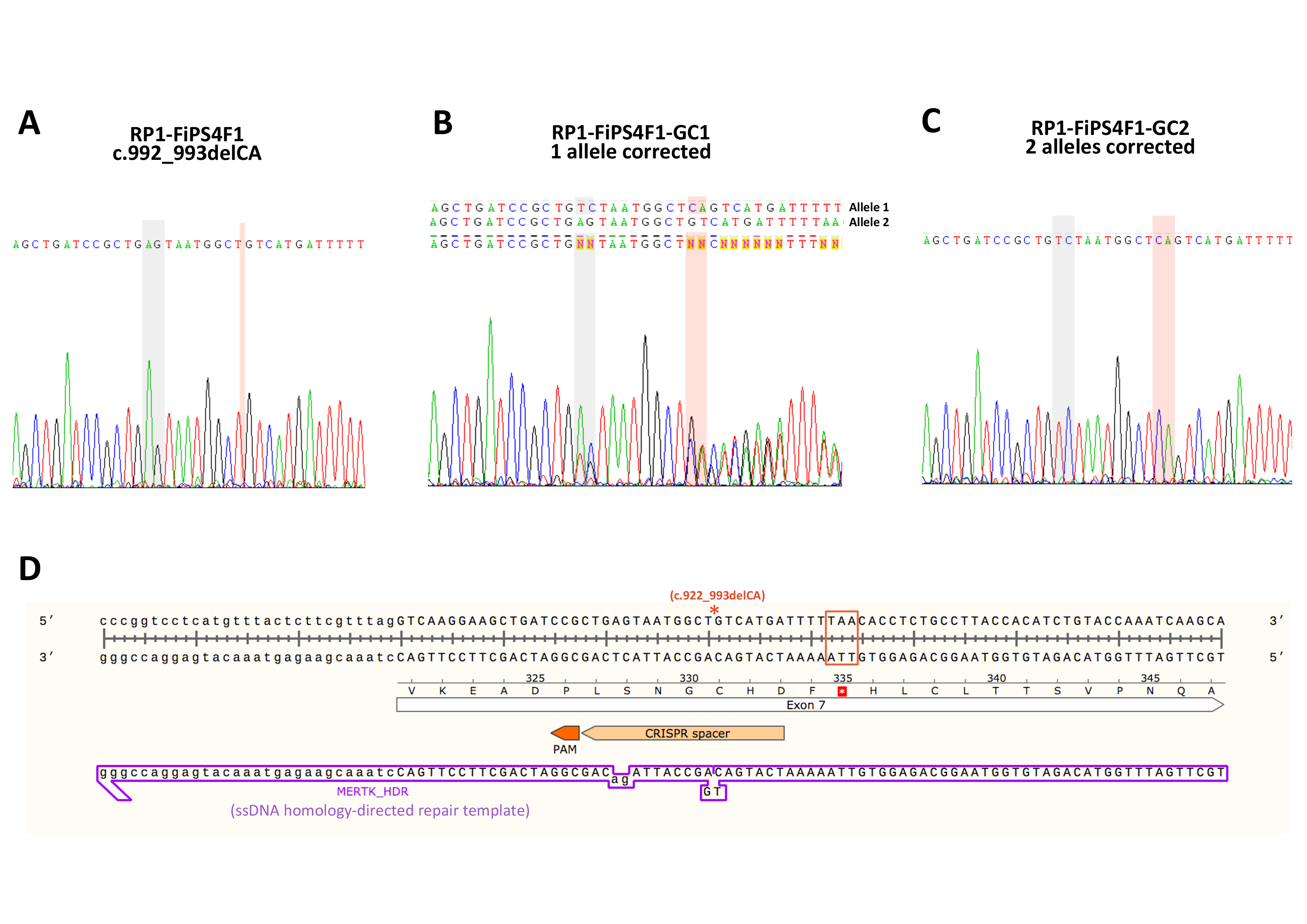

Supplement: Supplementary file 1 [file ijms-22-02092-s001.zip › ijms-1090241/Supplementary Figure 1.tif]

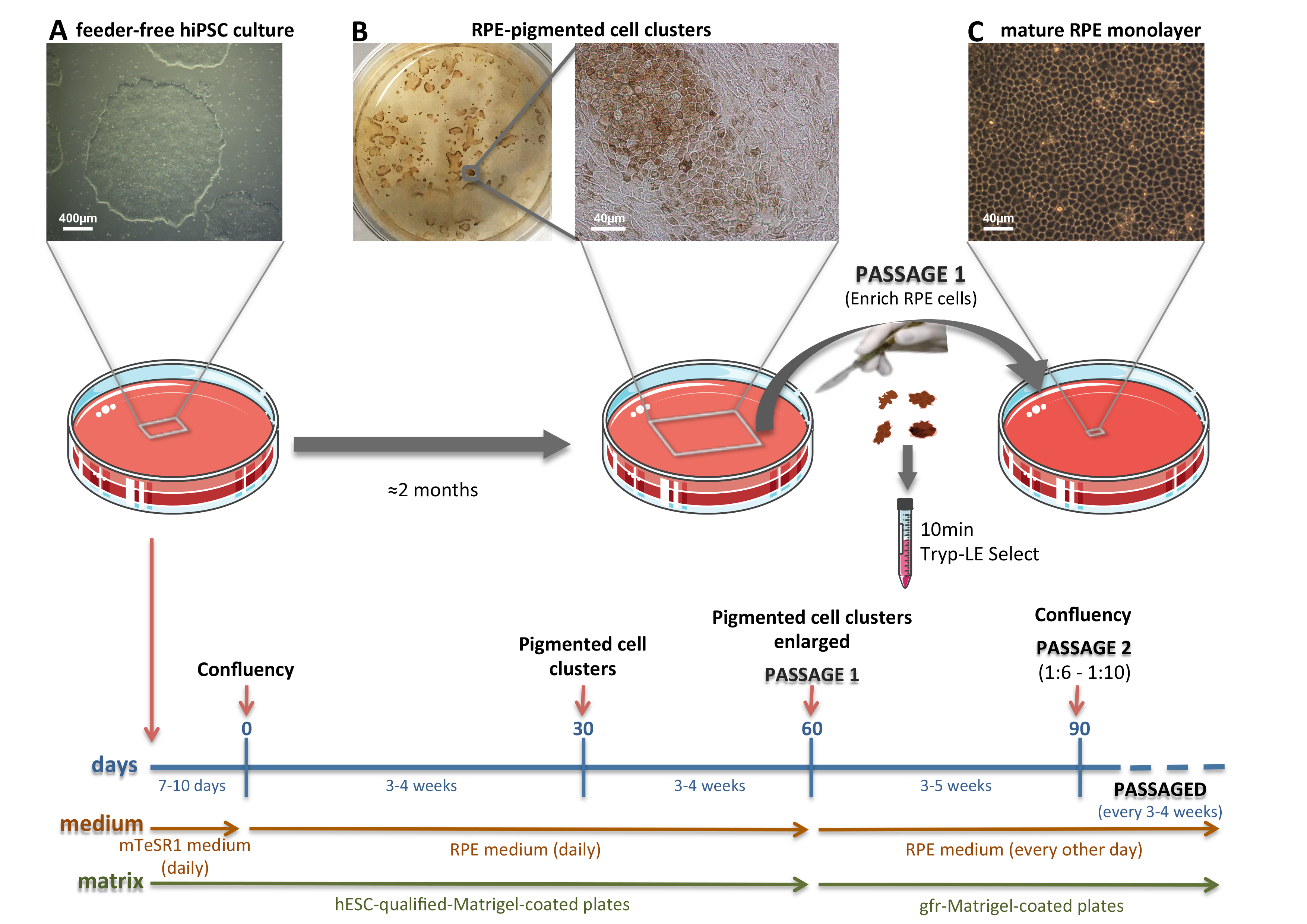

Supplement: Supplementary file 1 [file ijms-22-02092-s001.zip › ijms-1090241/Supplementary Figure 2.tif]

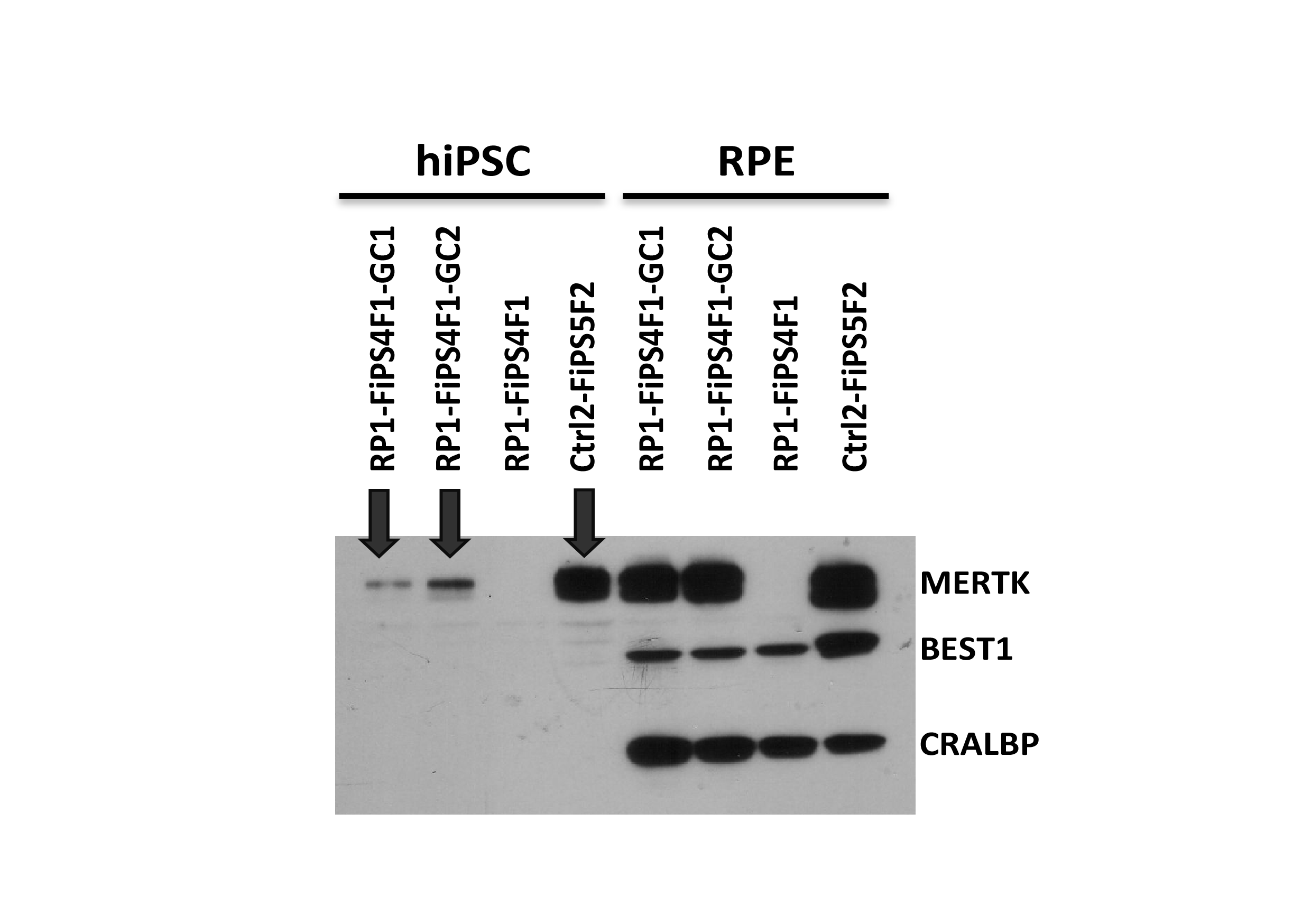

Supplement: Supplementary file 1 [file ijms-22-02092-s001.zip › ijms-1090241/Supplementary Figure 3.tif]
